# A dissipative particle dynamics study of the realignment of a nanodroplet of a nematic in a weak external magnetic field

**DOI:** 10.1140/epje/i2006-10080-6

**Published:** 2007-05-14

**Authors:** Y. K. Levine, A. Polimeno

**Affiliations:** 1 Section for Computational Biophysics, Department of Physics and Astronomy, Ornstein Laboratory, P.O. Box 80.000, 3508 TA Utrecht, The Netherlands; 2 Dipartimento di Scienze Chimiche, Università di Padova, Via Loredan 2, 35131 Padova, Italy

**Keywords:** 61.30.Cz Molecular and microscopic models and theories of liquid crystal structure, 61.43.Bn Structural modeling: serial-addition models, computer simulation

## Abstract

We present a dissipative particle dynamics (DPD) approach for simulating the realignment of a nematic nanodroplet suspended in an isotropic fluid following a switch in the direction of an applied external magnetic field. The interaction of the mesogens with the external field is weak relative to the inter-molecular interactions. The simulations were used to investigate the way orientational equilibrium is re-established. The results reveal that the realignment process of the nanodroplet is consistent with its fluid structure. The reorientation of the nanodroplet as a whole is found to be caused by an internal structural rearrangement rather than a coherent rotation of the centres of mass of the mesogens about the centre of the nanodroplet. The switch in the field direction furthermore is found to induce a transient spatial variation in the orientational order of the long axes of the mesogens: the orientational order parameters decreases on moving from the core of the nanodroplet to the surface in contact with the isotropic environment. The results highlight differences between the time evolution of the orientation of the long molecular axes in the field and the rotations of the centres of mass of the mesogens about the centre of the nanodroplet.

## 1 Introduction

Dissipative particle dynamics (DPD) [1–5] is a computational method based on the concept of “matter particles”, each representing a cluster of atoms or mesogens, interacting via soft potentials and subject to dissipative and fluctuating forces. Thus polymeric systems can be described by simple particle-spring models with long chains represented by 20 to 50 particles [4]. The technique was originally developed for describing isotropic phases with the important advantage of providing a way of describing the qualitative behaviour of a fluid system at relatively long times. A dissipative particle dynamics description does not provide a picture of the static and dynamic properties of the oriented fluid phases in terms of molecular properties. However, it affords a rationalisation of complex dynamic processes in ordered liquid phases in physical terms, which can be complementary to more detailed, atomic-based simulation tools. This is expected to be particularly true when considering classical rheological experiments monitoring the distribution of the director orientation patterns in nematic and smectic phases in the presence of mechanical or field-induced stresses, which are known to exhibit dynamic phenomena on different time scales, and moreover to be highly dependent on the system geometry, order parameter as well as field intensity.

We have demonstrated [6] that orientational and translational order can be induced with the “soft” interaction potential employed in conventional DPD algorithms. The systems used consisted of chains of particles whose flexibility has been hindered by an externally imposed harmonic potential. The computational effort is relatively modest compared to that demanded by other simulation techniques, thus establishing DPD as a convenient coarse-graining technique for numerical simulations of liquid-crystalline phases.

We have here used the DPD approach to simulate a non-equilibrium situation, namely the realignment of a nanodroplet of a nematic liquid crystal suspended in an isotropic fluid under the effect of an external magnetic field. Experiments utilizing such systems have been reported in [7–9]. The interaction of the mesogens with the external field is weak relative to the inter-molecular interactions.

The paper deals separately with the rotations of the centres of mass of the mesogens about the centre of the nanodroplet and realignment of the long axes about the centres of the mesogens. We furthermore investigate the spatial inhomogeneities in the properties of the nanodroplet during the realignment process. We find that the overall orientation of the nanodroplet in the field is consistent with a fluid structure and caused by an internal structural rearrangement rather than a coherent rotation of the centres of mass of the mesogens about its centre. The switch in the field direction furthermore induces a transient spatial variation in the orientational order of the long axes of the mesogens: the orientational-order parameters decreases on moving from the core of the nanodroplet to the surface in contact with the isotropic environment. Nonetheless, realignement process of the sample-averaged orientational-order parameters can be well described by the behaviour of a reorienting rigid monodomain nematic sample.

## 2 Dissipative particle dynamics simulations of ordered phases

### 2.1 Dissipative particle dynamics of nematic mesogens

The DPD methodology for the simulation of orientationally ordered phases has been described in detail previously [1–5] and will only be summarised here. The equations of motions governing the behaviour of particles of identical mass are

$$\eqalign{ & {\rm{d}}{{\rm{r}}_i} = {{\rm{v}}_i}{\rm{d}}t, \cr & m \cdot {\rm{d}}{{\rm{v}}_i} = ({{\rm{f}}_C} + {{\rm{f}}_D} + {{\rm{f}}_R}){\rm{d}}t \cr} $$,

where **r**_*i*_ and **v**_*i*_ are the position and velocity of the *i*-th particle, respectively. The conservative force, **f**_*C*_, arises from a pair-wise potential, while **f**_*D*_ and **f**_*R*_ represent the effective dissipative and fluctuating forces, respectively. The potential giving rise to the conservative forces acting on identical particles has the form of a sticky soft interaction:

$${U_C}({r_{ij}}) = \left\{ {\matrix{ {{{a\varepsilon _{ij}^{rep}{r_c}} \over 2}{{\left( {1 - {{{r_{ij}}} \over {{r_c}}}} \right)}^2},} \hfill & {{r_{ij}} < {r_c},} \hfill \cr {{{ - a\varepsilon _{ij}^{att}{r_c}} \over 2}{{\left( {1 - {{{r_{ij}}} \over {{r_c}}}} \right)}^2},} \hfill & {{r_{ij}} < {r_c} + \delta r,} \hfill \cr {0,} \hfill & {{r_{ij}} > {r_c} + \delta r,} \hfill \cr } } \right.$$

where *a* is the force parameter, setting the overall repulsion strength between particles, *ɛ*_*ij*_^*rep*^ and *ɛ*_*ij*_^*att*^ modulate the repulsive and attractive interactions, respectively, *r*_*c*_ is the cut-off radius of the repulsive interaction and *r*_*ij*_ the distance between the particles. The attractive interaction corresponding to a sticky potential operates in a thin shell of width *δ* *r* around the particle. We have here set *δ* *r* = 0.1*r*_*c*_. Our choice for the form of the attractive interaction has been made in the spirit of the DPD framework though other forms can be used [4]. The conservative force f_*C*_ acting on the *i*-th particle is then simply given by

$${{\rm{f}}_C}({r_{ij}})\left\{ {\matrix{ {a\sum\limits_j {\varepsilon _{ij}^{rep}\left( {1 - {{{r_{ij}}} \over {{r_c}}}} \right),} } \hfill & {{r_{ij}} < {r_c},} \hfill \cr { - a\sum\limits_j {\varepsilon _{ij}^{rep}\left( {1 - {{{r_{ij}}} \over {{r_c}}}} \right),} } \hfill & {{r_{ij}} < {r_c} + \delta r,} \hfill \cr {0,} \hfill & {{r_{ij}} > {r_c} + \delta r,} \hfill \cr } } \right.$$

where **^r**_*ij*_ is the unit inter-particle. The dissipative force **f**_*D*_ is defined as a quantity proportional to the relative velocity between the particles,

$${{\rm{f}}_D} = - \gamma \sum\limits_j {w_C^2({r_{ij}})} {\rm{v}}_{ij}^{||}$$.

Here **v**_*ij*_^‖^ is the projection of the relative velocity on the vector **^r**_*ij*_ and ${w_C}\left( {{r_{ij}}} \right) = 1 - {r_{ij}}/{r_c}$ when ${r_{ij}} < {r_c} + \delta r$ and zero elsewhere. The dissipation strength is determined by *γ*, and the corresponding effective random force is defined as ${{\rm{f}}_R} = \sigma \xi (t){w_C}({r_{ij}}){{{\rm{\hat r}}}_{ij}}/\sqrt {{\rm{d}}t}$, where *ξ*(*t*) is a white-noise random term. A fluctuation-dissipation relation holds for the parameters of the dissipative and random forces, *σ* ^2^ = 2*γκ*_*B*_ *T*.

In the simulations below we make use of reduced units where *r*_*c*_, *m* and *a* are the units of length, mass and force, respectively. The other units are defined in Table 1.

**Table 1:** Definition of reduced units.

ρ* = *ρr*_*c*_^3^	Reduced density
T* = *κ*_*B*_*T/ar*_*c*_	Reduced temperature
E* = *E/ar*_*c*_	Reduced energy
κ* = *kr*_*c*_/*a*	Reduced elastic constant
t* = (*a/mr*_*c*_)^3/2^*t*	Reduced time
γ* = *γ*(*r*_*c*_/*ma*)^1/2^	Reduced friction
σ*^2^ = 2T*γ*	Reduced fluctuation-dissipation relation
ɛ* = *ɛ/ar*_*c*_	Reduced energy

Each mesogenic unit in the simulation is a chain of n particles linked by elastic forces given by the classical spring force ${{\rm{f}}_i} = - k*({r_{ij}} - {r_{eq}}){{{\rm{\hat r}}}_{ij}} = - {{\rm{f}}_j}$, with k* the reduced spring constant, *r*_*ij*_ the distance between the two joined particles and *r*_*eq*_ the equilibrium particle-particle distance. In addition, an elastic force with an elastic constant *k*_e_^*^ is introduced between the first and last particles: ${{\rm{f}}_1} = - k_e^*\left[ {{r_{1n}} - (n - 1){r_{eq}}} \right]{{{\rm{\hat r}}}_{1n}} = - {{\rm{f}}_n}$. This force primarily determines the linear extent of the mesogenic unit and random coils are obtained when it is omitted.

A spherical simulation box of radius *R* centred at the origin was used in this study. The mesogenic units were confined to the box by the application of a short-range external radial force to each particle, $i:{{\rm{f}}_{i,sphere}} = k_{in}^*\exp [ - 2.5(R - {r_i})]{{{\rm{\hat r}}}_i}$, where **^r**_*i*_ is the unit position vector of the particle with respect to the origin of the simulation sphere.

The equations of motion are solved numerically using the standard Verlet algorithm coupled with a thermostat rescaling the velocities. Periodic boundary conditions were not applied as we are here dealing with a single nanodroplet in the simulation sphere. A linked-cell algorithm [10] was implemented.

### 2.2 Realignment of nematics in an external field

We shall now consider the alignment of the model mesogens in the nandroplet under the influence of an external field. The applied field acts on the two terminal particles of the mesogenic unit (*i.e.* on the end-to-end vector of the mesogen). The interaction used here corresponds to the application of an external magnetic field and takes the form of an interaction between a field and an induced dipole:

$$E_{ext}^*(t*) = - \varepsilon _{ext}^*{P_2}\left[ {{v_i}(t*) \cdot {{\rm{H}}_{ext}}} \right]$$,

where *ɛ*_ext_^*^ is the strength of the interaction and *ν*_*i*_(t*) is the unit end-to-end vector of the *i*-th mesogen and **H**_*ext*_ is the unit vector of the external field. In the work presented here, the external field lies in the *X Z* plane of the simulation sphere. The calculation of the forces from the potential is described in the appendix.

### 2.3 Characterisation of the nanodroplet shape

The shape of the nanodroplet is readily characterised by evaluating its tensor of inertia

$${I_{\alpha \beta }} = \sum\limits_{i = 1}^N {r_{\alpha ,i}^{cm}r_{\beta ,i}^{cm}/N} ,{\rm{ }}\alpha \beta \equiv x,y,z,$$

using the Cartesian components of the position vector of the centres of mass, **r**_*i*_^*cm*^, of the N mesogens relative to the centre of mass of the nanodroplet. The principal axes of inertia and their orientation in the laboratory are obtained from a diagonalisation of the matrix *I*_*αβ*_.

### 2.4 Orientation of the long axes of the mesogens in the simulation sphere

The rotational anisotropy of the long axes of the mesogens with respect to the fixed *Z*-axis is characterised by the orientational-order parameter *S*_*zZ*_(t*):

$${S_{zZ}}(t*) = \sum\limits_{\rm{i}} {{P_2}\left[ {\cos {\theta _i}(t*)} \right]/N} $$,

where *θ*_*i*_(t*) is the angle made by the end-to-end vector of the mesogen with the *Z*-axis at time t*. Similarly, we calculate the order parameters *S*_*zX*_(t*) and *S*_*zY*_(t*) of the end-to-end-vector relative to the *X*- and *Y*-axes, respectively. As the three parameters are not independent, but related by ${S_{zX}}(t*) + {S_{zY}}(t*) + {S_{zZ}}(t*) = 0$, we shall here only discuss the behaviour of the latter two order parameters.

### 2.5 Rotation of the centres of mass of the mesogens about the centre of the nandroplet during realignment

The whole-body rotation of the mesogens as the result of the switching of the applied field can be followed by calculating the angular excursion of their centres of mass about the three axes of the simulation sphere, *θ*_*X*_, *θ*_*Y*_ and *θ*_*Z*_. This can be achieved in two ways. The angles can be computed from the spherical components of the position vectors relative to the axes of the simulation sphere. This, however, requires numerical checks to take account of sudden changes in the angle of azimuth across the 0°/360° boundary. An equivalent, though simpler procedure which does not suffer from this makes use of the projections of the position vectors of the centre of mass on to the *ZY*, *XZ* and *XY* planes, respectively. The rotations are calculated by evaluating the vector products between the components at times t* and t*+dt* and making use of the fact that we are dealing with small angles so that sin *θ* ≈ *θ*. In this way we obtain the following expressions for time evolution of the rotation angles about the three fixed axes, *X, Y* and *Z* during the course of the simulation:

$$\eqalign{ & \left\langle {{\theta _X}(t* + {\rm{d}}t*)} \right\rangle = \left\langle {{\theta _X}(t*)} \right\rangle + \left\langle {{y^{cm}}(t* + {\rm{d}}t*){z^{cm}}(t*) - {y^{cm}}(t*){z^{cm}}(t* + {\rm{d}}t*)} \right\rangle R({y^{cm}},{z^{cm}}), \cr & \left\langle {{\theta _Y}(t* + {\rm{d}}t*)} \right\rangle = \left\langle {{\theta _Y}(t*)} \right\rangle + \left\langle {{z^{cm}}(t* + {\rm{d}}t*){x^{cm}}(t*) - {z^{cm}}(t*){x^{cm}}(t* + {\rm{d}}t*)} \right\rangle R({x^{cm}},{z^{cm}}), \cr & \left\langle {{\theta _Z}(t* + {\rm{d}}t*)} \right\rangle = \left\langle {{\theta _Z}(t*)} \right\rangle + \left\langle {{x^{cm}}(t* + {\rm{d}}t*){y^{cm}}(t*) - {x^{cm}}(t*){y^{cm}}(t* + {\rm{d}}t*)} \right\rangle R({x^{cm}},{y^{cm}}), \cr & R(\alpha ,\beta ) = 1/\sqrt {\left[ {{\alpha^2}(t*) + {\beta^2}(t*)} \right]\left[ {{\alpha^2}(t* + {\rm{d}}t*) + {\beta^2}(t* + {\rm{d}}t*)} \right]} ; \cr & \alpha ,\beta = {x^{cm}},{y^{cm}},{z^{cm}} \cr} $$.

The angled brackets denote an average over the mesogens in the nanodroplet.

## 3 Results of the simulations

### 3.1 System parametrisation

The internal conformation of a model mesogenic unit is characterized by 4 parameters: the number of particles n, the two elastic spring constants, k* and *k*_e_^*^ and the equilibrium distance between joined particles, *r*_*eq*_. We have previously shown that on using identical mesogenic units each containing 10 particles with *r*_*eq*_ = *r*_*c*_, *ɛ*_ij_^*rep^ = 24, *ɛ*_ij_^*att^ = 0, k* = 100 and *k*_e_^*^ = 500, at a reduced density ρ* = 0.8 the system forms a nematic phase in the reduced temperature range 0.25 ≤ T* ≤ 0.5 [6].

The current simulations make use of a nanodroplet of a nematic phase suspended in an isotropic environment. The isotropic environment consists of mesogenic units in a random-coil configuration, that is for these mesogens *k*_e_^*^= 0. We found that this prescription avoided the numerical instabilities reported in [5] for simple fluids of spheres. The radius of the nanodroplet was taken to be 0.7*R*, with *R* the radius of the spherical simulation box. On taking *R* = 143, the system configuration consisted of 36786 stretched mesogens in the nanodroplet and 69684 random coils forming the isotropic environment. Under this condition the external confining force, k*_in_ = 24, only acts on the random coils at every stage of the simulation run.

Time steps dt* = 0.04 were used and the integration scheme yielded temperature fluctuations of less than 0.25% in the equilibrated state. A thermostat was applied every 5 time steps during the simulation run.

We were unable to produce a stable nanodroplet using purely repulsive potentials and therefore described the particles as sticky spheres. To this end, the strengths of the inter-particle interactions between mesogens of the same configuration were taken to be *ɛ*_ij_^*rep^ = 24 and *ɛ*_ij_^*att^ = 60, while those between particles in the stretched and randomcoil configurations (in the nematic and isotropic phases, respectively) were *ɛ*_ij_^*rep^ = 60 and *ɛ*_ij_^*att^ = 0. This choice of parameters produced sufficient surface tension between the nanodroplet and its isotropic environment to suppress the exchange of mesogens between the two phases. We emphasise, however, that we did not carry out an exhaustive search of parameter space and that consequently other combinations of values may prove to be as effective.

**Fig. 1:**
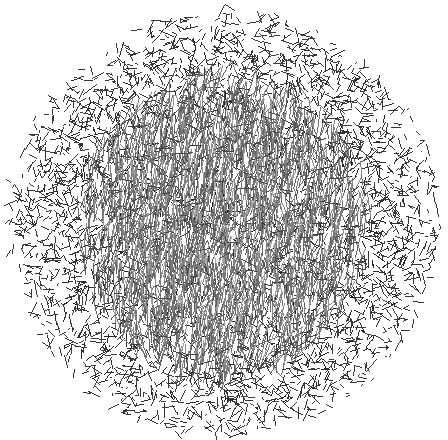
Image of the initial system configuration, T* = 0.325, with the *X Z* plane of the simulation sphere in the foreground. The image shows the end-to-end vectors of 15000 mesogenic units chosen at random.

The system configuration produced in this way was found to be stable in the reduced temperature range 0.25 ≤ T* ≤ 0.45. The nanodroplet underwent a nematic-isotropic transition at the upper limit of this interval. A typical image of the system at T* = 0.325 is shown in Figure 1. The image sets out the spatial positions of the terminal particles of 15000 mesogens selected at random. The particles are connected by a virtual bond and the image shows a view projected on the *X Z* plane of the simulation sphere.

Realignment experiments on nematic phases are usually carried out with magnetic field of up to about 10T [11,12]. Under these conditions the interaction of the nematogens with the external field is much weaker than the inter-molecular interactions in the system. We shall here emulate this situation and choose *ɛ*_ext_^*^ to be as small as practically possible. We have found that on taking *ɛ*_ext_^*^ = 0.1, the orientational energy in the applied field amounts to about 3% of the inter-molecular energy. With this value of *ɛ*_ext_^*^ we find that the equilibrium value of *S*_*zZ*_ increases from 0.76 to 0.84 on applying the orienting field, indicating that the external interaction only causes a small change in the intrinsic system properties. This change was found to be due entirely to an alignment of the end-to-end vectors of the mesogens in the field. The dimensions of the mesogens are not affected by the presence of the external field. We note here that the internal elastic energy of a model mesogen is 3 orders of magnitudes larger than the orientational interaction.

In order to check whether the realignment processes depend on the value of the field, we carried out a single simulation run with *ɛ*_ext_^*^ = 0.02, 5 times smaller than that used in the results presented below. Now the equilibrium value of *S*_*zZ*_ increased from 0.76 to 0.775 on applying the orienting field.

The starting point of the simulations was a thermally equilibrated system configuration of mesogens with the external field applied along the *Z*-axis of the simulation sphere. We note that the field only acts on the stretched mesogens constituting the nanodroplet.

At time t* = 0, the external field was switched in the *X Z* plane to a different orientation at an angle *θ*_*ext*_ relative to the *Z*-axis. The properties of the system were then monitored for t* ≤ 5000 during the return to orientational equilibrium along the direction of the field. The applied field was switched by angles of 30°, 45°, 60°, 75° and 90°.

The results of the simulations reported below for a switch angle of 60° were virtually identical with those obtained with *ɛ*_ext_^*^ = 0.02 provided the time axis was scaled by a factor of 5 as expected theoretically. This is a strong indication that the mechanism of alignment is independent of the field value for our choice of parameters.

Finally, it is important to emphasise that the latter case, a switch of the applied magnetic field by 90°, is a special case [12]. The field-mesogen interaction, equation (5), has an extremum when the angle between the field and the long axis of a mesogen is 90°. Consequently, the external torque working on the mesogen works in a clockwise direction when the angle is less than 90° and counter-clockwise when it is greater than 90°. The torque changes direction as the long axis of the mesogen swings through 90° as the result of orientational fluctuations and thus enhances the effect of the fluctuations. This degeneracy should produce two counter-rotating mesogen populations, resulting in a symmetric distribution of rotations about the *Y*-axis of the simulation sphere. Furthermore, anomalous changes in the internal structure of the nanodroplet are expected to be observed during the realignment process.

### 3.2 Equilibrium properties

**Fig. 2:**
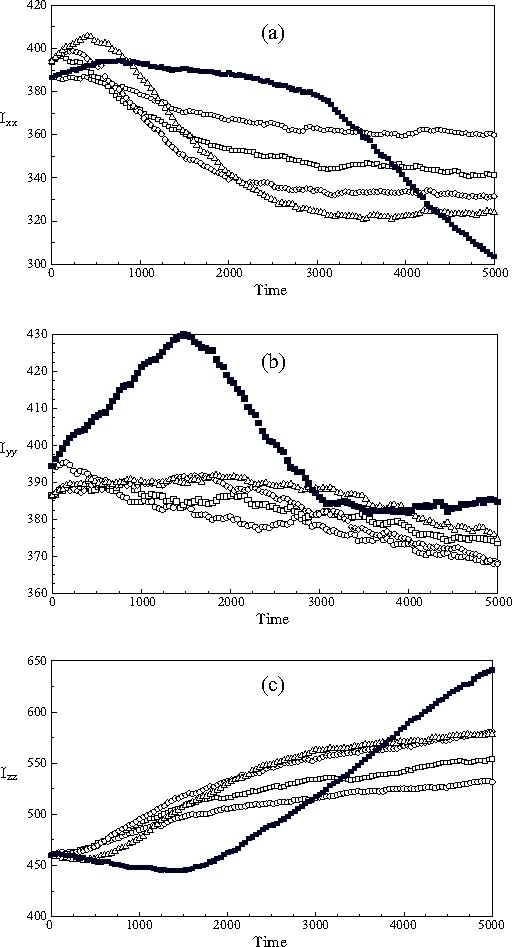
The time course of the principal eigenvalues of the moment-of-inertia tensor of the nanodroplet: (a) *I*_*xx*_, about the x-axis of the nanodroplet; (b) *I*_*yy*_, about the *y*-axis of the nanodroplet and (c) *I*_*zz*_, corresponding to the long *z*-axis of the nanodroplet: (∘) 30° switch; (□) 45° switch; (◊) 60° switch; (▵) 75° switch and ▪ 90° switch.

The eigenvalues (principal values) of the moment-of-inertia tensor of the nanodroplet were calculated to be 386.5, 393.4 and 460.2 about the *x*-, *y*- and *z*-axis of the nanodroplet, Figures 2. The equilibrium shape of the nanodroplet is thus essentially a prolate sphere with its long axis oriented along the Z-axis of the simulation sphere.

The spherical nanodroplet was found to be spatially homogeneous except for an outer shell in contact with the isotropic environment. The heterogeneity in the properties of the nanodroplet can be monitored by dividing it into a series of shells. To this end we calculated the position vector of the centre of mass of each mesogen, **r** ^*cm*^, and binned it according to the following scheme:

**Table:** 

Core			|**r** ^*cm*^|	≤	20	(2851 mesogens),
Shell 1	20	<	|**r** ^*cm*^|	≤	30	(6636 mesogens),
Shell 2	30	<	|**r** ^*cm*^|	≤	40	(13187 mesogens),
Outer shell			|**r** ^*cm*^|	>	40	(14116 mesogens).

The corresponding values of the order parameter *S*_*zZ*_ were found to be 0.865, 0.871, 0.867 and 0.780. The latter value obtained for the outer shell is lower than the rest as a result of the constraints on the alignment of the long axes of the mesogens by the curved surface of the nanodroplet.

### 3.3 Shape of the nanodroplet during alignment

The principal values of the moment-of-inertia tensor of the nanodroplet vary smoothly by around 15% during the realignment process following the switching of the magnetic field by less than 90°, Figures 2. However, anomalous changes are observed following a 90o switch in the direction of the field, as expected from the degeneracy of the field-mesogen interactions immediately after the switching of the field. Importantly, we note that the nanodroplet does not reach an equilibrium shape during the simulation run.

It can be seen that the limiting values of the principal moments of inertia about the *x*- and *z*-axes of the nanodroplet reached at long time, t* > 3000, are significantly different from their initial, equilibrium, values. This indicates that the internal configuration of the nanodroplet changes irreversibly during the realignment process. Nevertheless, the principal moment of inertia about the *y*-axis of the drop is essentially constant, and this is consistent with the switching of the field in the *Z X* plane of the simulation sphere. Importantly, we find that the magnitude of the change increases on increasing the switching angle of the field, Figures 2. It is important to note that in our simulations the time scale of translational diffusion is comparable to that of the rotational realignment processes. We believe that the change of the nanodroplet shape during the realignment process can be ascribed to the combination of the random distribution of the centre of mass of the mesogens and their anisotropic translational diffusion.

**Fig. 3:**
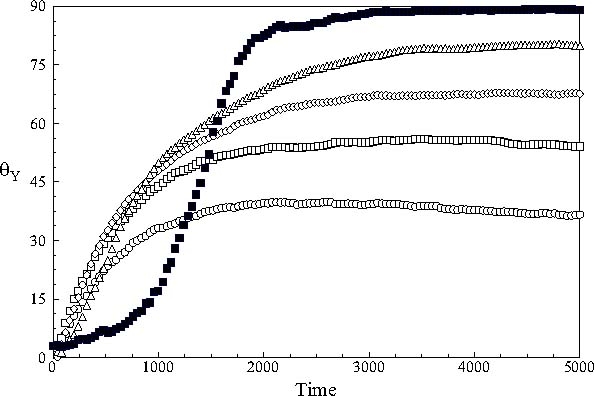
The angle of rotation of the corresponding eigenvector about the fixed Y -axis of the simulation sphere: (ℴ) 30° switch; (□) 45° switch; (◊) 60° switch; (▵) 75° switch and (▪) 90° switch.

An estimate of the magnitude of the whole-body rotation of the nanodroplet in the *X Z* plane away from the *Z*-axis of the simulation sphere towards the field may be obtained from the time dependence of the eigenvector corresponding to the long axis of the nanodroplet, Figure 2c, on assuming that the changes of shape are negligible. The angles of rotation of this eigenvector about the *Y*-axis of the simulation sphere obtained in this way are shown in Figure 3 for all the cases studied. The limiting values reached at long times are found to be close to the switching angle applied.

We shall now address the question as to whether the nanodroplet rotates as a rigid entity and if the changes of the overall shape found here are due to spatial inhomogeneities in its configuration.

### 3.4 Rotation of the centres of mass of the mesogens about the centre of the nanodroplet

**Fig. 4:**
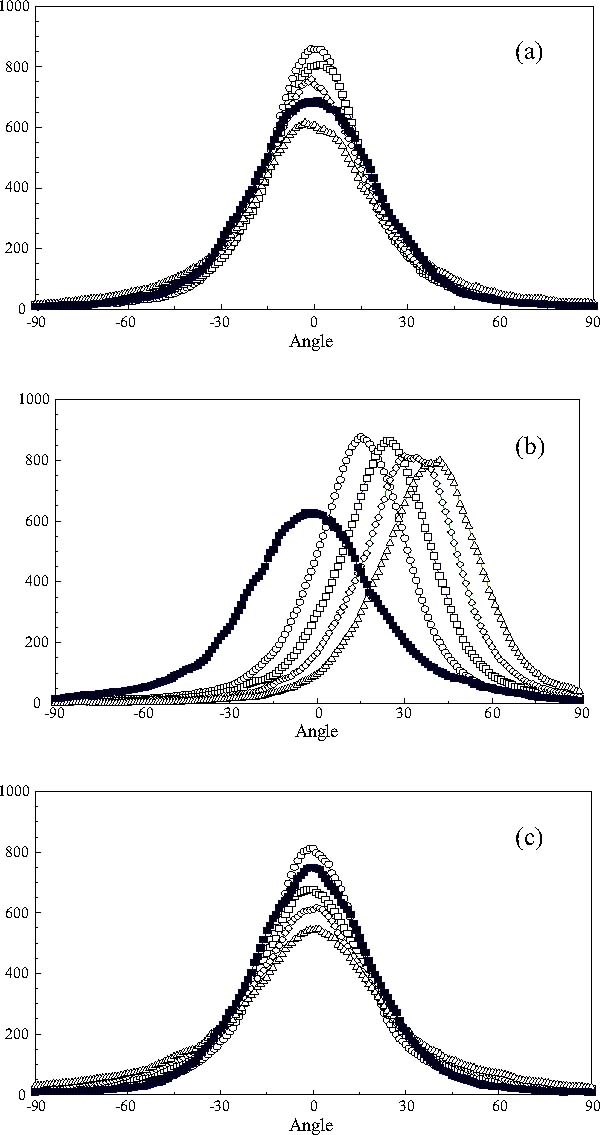
The distribution of the angles of rotation of the centres of masses of the mesogens about the fixed axes of the simulation sphere between the switch of the field direction at t* = 0 and t* = 5000: (a) rotation about the fixed *X*-axis; (b) rotation about the fixed *Y*-axis and (c) rotation about the fixed *Z*-axis; (□) 45° switch; (◊) 60° switch; (▵) 75° switch and (▪) 90° switch.

The distributions of the rotation angles of the centres of mass of the mesogens at t* = 5000 are shown in Figures 4 in the interval −90° ≤ *θ* ≤ 90°. The angles for the rotations about the fixed *X*- *Y*- and *Z*-axes were calculated from (8) during the simulation run. The results show that the mesogens essentially undergo a stochastic rotation about the *X*- and *Z*-axes, Figures 4a and c and that the distribution broadens somewhat with increasing switching angle. We find that the average rotation about these axes is about 2°–4°. A concerted rotation is only observed for the rotation about the *Y*-axis in line with expectations. Again broad distributions are observed, but their peaks—and average values— are significantly smaller than the corresponding switching angle. The anomalous behaviour expected for the degenerate situation of a 90° switch is indeed observed in the results. We find that the centre of the distribution of rotations is close to *θ*_*Y*_ = 0°, and that in this case the centres of mass of the mesogens undergo stochastic rotations about all the fixed axes. This finding contrasts markedly with the observation, Figure 3, that the long axis of the nanodroplet rotates by 90° during the simulation run. These findings are in accord with the theoretical expectations set out above.

**Fig. 5:**
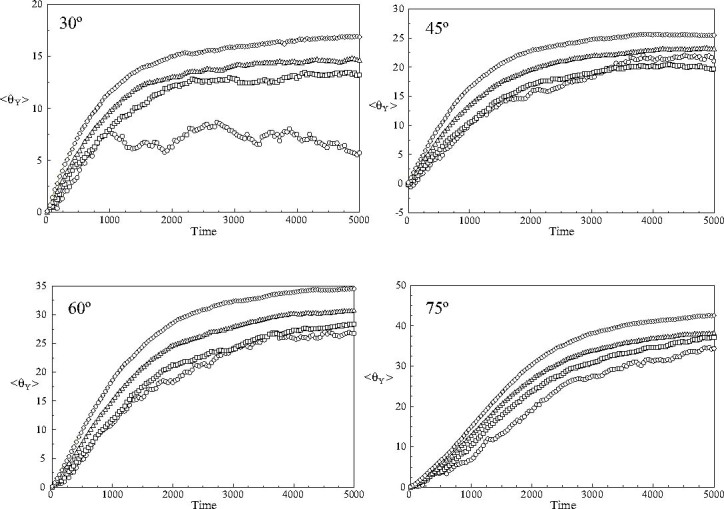
The rotations of the centres of mass of the mesogens about the fixed Y -axis of the simulation sphere in the core of the nanodroplet and the three outer shells for switching angles of 30°, 45°, 60° and 75°: (ℴ) core; (□) first shell; (▵) second shell and (◊) outer shell.

The averaged rotations of the centres of mass of the mesogens in the core of the nanodroplet and the three spherical shells about the *Y*-axis of the simulation sphere are shown in Figure 5. It can be clearly seen that the nanodroplet exhibits a gradient of rotational motions: mesogens at the core undergo significantly smaller rotations about the *Y*-axis than mesogens in the outer shell. Moreover, the average rotation of the centres of mass of the mesogens about the centre of the nanodroplet is considerably smaller than the switching angle of the applied field in every region. This strongly indicates that the rotation of the long axis of the moment-of-inertia tensor of the nanodroplet, Figure 3, occurs as the result of a structural rearrangement of mesogens in the nanodroplet rather than a coherent rotation of the centre of mass of the mesogens characteristic of the rotation of a rigid nanodroplet.

### 3.5 Orientational distributions of the long axes of the mesogens during realignment

**Fig. 6:**
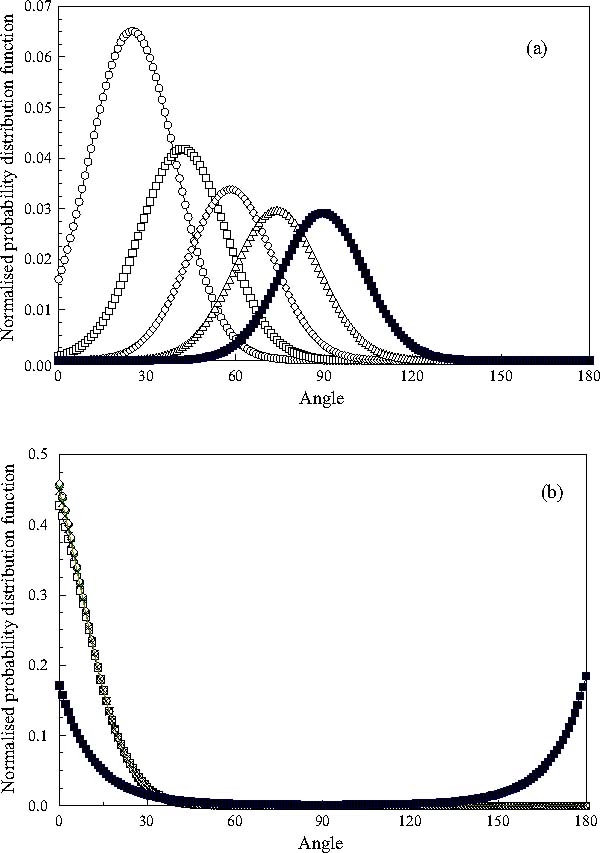
The normalized orientational probability distribution functions with respect to the position of the applied field immediately after a switch (a), and at the end of the simulation run (b): (ℴ) 30° switch; (□) 45° switch; (◊) 60° switch; (▵) 75° switch and (▪) 90° switch.

The normalised orientational probability distribution functions of the long molecular axes of the mesogens immediately following the switch in the direction of the applied field and those found at the end of the simulation time t* = 5000 are shown in Figures 6a and b, respectively. The initial distribution functions are clearly broad and unimodal. However, only the distribution functions for the 75° and 90° switching angles exhibit a significant probability of orientations of the long axes of the mesogens at angles larger than 90° relative to the field. The final distribution functions are also unimodal, Figure 6b, and identical for all cases except that of a 90° switch of an applied field. It thus appears that the width of the initial orientational distribution functions and the fluctuations of the orientations of long axes of the mesogens induced by the DPD algorithm used here are not sufficient to tilt a significant population of molecular axes to angles larger than 90° for switching angles *θ*_*ext*_ ≤ 75°. Consequently, two counter-rotating orientational populations are only observed for a switching angle of 90°. Moreover, we note that the normalised orientational probability distribution functions shown in Figure 6b are virtually identical with those observed at equilibrium.

The realignment process of the long axes of the mesogens in the nanodroplet can be monitored in more detail by calculating the temporal behaviour of the orientation order parameters *S*_*zY*_ (t*) and *S*_zZ_(t*) following a switch in the orientation of the applied field. The order parameter *S*_*zY*_(t*) monitors the excursions of the long axes of the mesogens out of the *X Z* plane during the realignment process. The order parameter *S*_*zZ*_(t*) on the other hand, monitors the reorientation of the distribution of mesogen axes from the *Z*-axis at time t* = 0 towards the field. Again, we shall evaluate the order parameters separately for the core of the nanodroplet and the three outer shells.

**Fig. 7:**
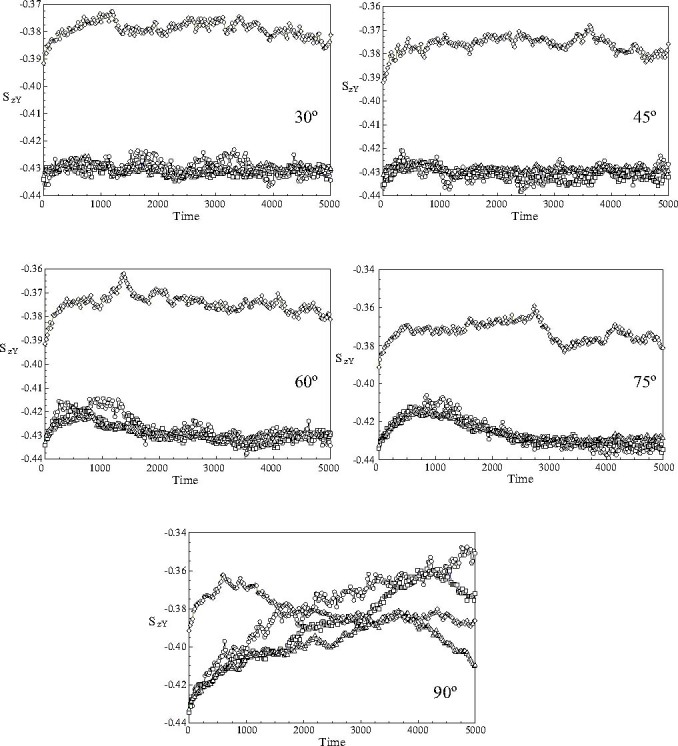
The time evolution of the order parameter *S*_*zY*_(t*) of the component of the long axes of the mesogens along the fixed *Y*-axis of the simulation sphere in the core of the nanodroplet and the three outer shells following the application of 30°, 45°, 60°, 75° and 90° switching angles: (ℴ) core; (□) first shell; (▵) second shell and (◊) outer shell.

The behaviour of the order parameters *S*_*zY*_ (t*) are shown in Figure 7. It can be seen that for all the switching angles studied here, the order parameters are negative and close to the value of −0.5 expected for a perfect alignment of the mesogen axes in the *X Z* plane. The order parameter for the outer shell of the nanodroplet is found to be significantly higher (corresponding to lower order) than those found in the bulk of the nanodroplet. The results clearly show that the order parameters monitored following a switch with 30° or 45° are essentially constant during the realignment process. In marked contrast, significant changes are observed for t* < 2000 at switching angles of 60° and 75°. Interestingly, the values of the order parameters at the end of the simulation run are close to the equilibrium values. An anomalous behaviour is observed following a 90° switch of the magnetic field, Figure 7, where a complex time evolution in the values of the time order parameters in the core and three shells are observed.

**Fig. 8:**
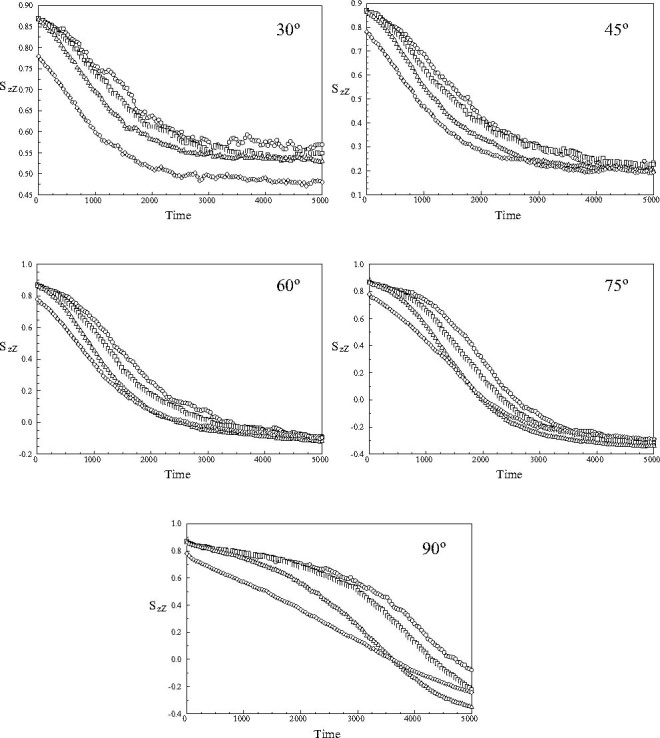
The time evolution of the order parameter *S*_*zZ*_(t*) of the component of the long axes of the mesogens along the fixed *Z*-axis of the simulation sphere in the core of the nanodroplet and the three outer shells following the application of 30°, 45°, 60°, 75° and 90° switching angles: (ℴ) core; (□) first shell; (▵) second shell and (◊) outer shell.

The time course of the values of the order parameters *S*_*zZ*_(t*) in the different regions of the nanodroplet is shown in Figure 8. It can be seen that the switching of the applied field induces a significant spatial gradient in the orientational order from the core to the outer shell during the realignment process. Nonetheless, a homogeneous distribution is recovered at long times, t* > 4000 except for the cases of switches of the applied field by 30o and 90o, where the inhomogeneity persists at the end of the simulation run. For all other angles the values of the order parameters of the long axes of the mesogens calculated at the end of the simulation run with respect to the field are close to the initial equilibrium values. Thus, an orientational equilibrium is re-established along the applied field. This is in contrast to the mechanical properties described in Section 3.4.

The spatial inhomogeneity in the orientational order found in our simulations is not revealed experimentally, where commonly order parameters averaged over all the mesogens in the sample are monitored. We shall therefore now consider the behaviour of the sample-averaged order parameters and address the question as to whether they can be described by the analytical approaches used to describe experimental data. For simplicity we shall only consider the torque-balance model [13,14] for the realignment of a rigid monodomain nematic sample in a magnetic field. The equation of motion of the director of the sample is given by

$$\tan \beta (t) = \tan {\beta _0}\exp ( - t/\tau )$$

where *β*(*t*) is the angle made between the director and the external field at time t following an initial switch by an angle *β*_0_ at time *t* = 0. We shall now assume that the orientational distribution of the long axes of the mesogens retains its initial unimodal form during the realignment process. Under this assumption we can express the orientation of any mesogen in the nanodroplet in terms of two successive rotational transformations: i) a rotation from the fixed Z-axis to the instantaneous axis of symmetry of the distribution and ii) a rotation from the instantaneous symmetry axis to the long axis of the mesogen. Transformation i) can then be used to characterise the behaviour of the simulated system as a monodomain.

The transformation properties of orientational-order parameters under rotations have been discussed in detail in the literature [15,16] and it is known that the order parameter *S*_*zZ*_(t*) calculated in the laboratory frame for a uniaxial distribution can be written as

$${S_{zZ}}(t*) = {P_2}\left[ {\cos {\theta _A}(t*)} \right]{S_{zZ}}{(t*)_0}$$,

where *S*_*zZ*_(t*)_0_ is the intrinsic order parameter of the distribution calculated relative to the symmetry axis, and *θ*_*A*_(t*), is the angle made by the symmetry axis with the fixed *Z*-axis at time t*. On setting *S*_*zZ*_(t*)_0_ = *S*_*zZ*_(t* = 0), we can extract the order parameter of the instantaneous orientation of the symmetry axis of the distribution from the normalised order parameter *S*_*z*_(t*)/*S*_*z*_(t* = 0). The angle *β*(t*), equation (9), between the director of the nematic monodomain and the applied magnetic field can be calculated since

$${S_{zZ}}(t*)/{S_{zZ}}(t* = 0) = 0.5(3{\cos^2}[{\beta _0} - \beta (t*)] - 1)$$.

**Fig. 9:**
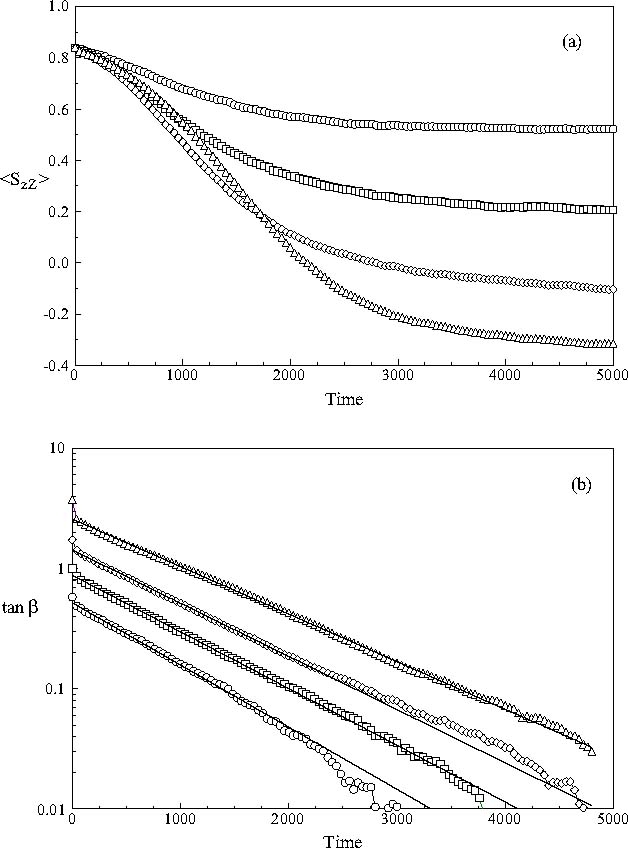
(a) The time evolution of the averaged order parameter 〈*S*_*zZ*_(t*)〉 of the component of the long axes of the mesogens in the nanodroplet along the fixed Z-axis of the simulation sphere following the application of 30°, 45°, 60° and switching angles: (ℴ) 30° switch; (□) 45° switch; (◊) 60° switch and (▵) 75° switch. (b) The time evolution of the angle *β* made between the director of the nanodroplet and the applied field at time t* following an initial switch by an angle *β*_0_. The angle was extracted from 〈*S*_zZ_(t*)〉 as described in the text. The continuous lines show the best fit to the torque-balance equation and the symbols used are as in (a).

The sample-averaged order parameter 〈*S*_*zZ*_(t*)〉 and the fit of the extracted angle *β*(t*) to equation (9) are shown in Figures 9a and b, respectively. Surprisingly, the angles obtained from the simulations exhibit a monoexponential behaviour over large portions of their decays. The decay constants however, vary almost linearly from 840 at *θ*_*ext*_ = 30° to 1095 at *θ*_*ext*_ = 75°. It thus appears that the results presented here for the sample-averaged orientation order of the long molecular axes of the mesogens is described well by the torque-balance equation, even though the detailed behaviour is difficult to reconcile with its underlying assumptions.

## 4 Conclusions

We have here presented a dissipative particle dynamics study of the realignment kinetics of a nematic nanodroplet suspended in an isotropic fluid following a switch in the direction of an applied field. The results reveal significant spatial inhomogeneities in the properties of the nanodroplet, consistent with its fluid structure. The reorientation of the nanodroplet as a whole is found to be caused by an internal structural rearrangement rather than a coherent rotation of the centres of mass of the mesogens about the centre of the nanodroplet. The switch in the field direction furthermore is found to induce a transient spatial variation in the orientational order of the long axes of the mesogens: the orientational-order parameters decrease on moving from the core of the nanodroplet to the surface in contact with the isotropic environment. These results highlight the differences in the reorientation of the long molecular axes about the centres of mass of the mesogens, and the rotations of the centres of mass about the centre of the nanodroplet.

## Appendix A

We shall here consider the interaction, equation (5), between the unit end-to-end vector of a mesogen, *ν* = {*Δx/r,Δy/r,Δz/r*} and a magnetic field applied in the *X Z* plane of the simulation box, **H**_*ext*_ = {*H*_*x*_, 0, *H*_*z*_}. Here $r = \sqrt {\Delta {x^2}\Delta {y^2} + \Delta {z^2}}$ and $\Delta x = {x_1} - {x_n},{\Delta _y} = {y_1} - {y_n}$ and $\Delta z = {z_1} - {z_n}$. The subscripts 1 and *n* denote the two terminal particles of the mesogen. On substituting these values into (5) we find

$$E_{ext}^*(t*) = - \varepsilon _{ext}^*{P_2}[{v_i} \cdot {{\rm{H}}_{ext}}] = - 1/2\varepsilon _{ext}^*[\Delta {x^2}H_x^2/{r^2} + \Delta {z^2}H_z^2/{r^2} + 2\Delta x\Delta z{H_x}{H_z}/{r^2}] - 1/2$$

.

In order to calculate the force due to this potential, *F* = −∇*E*_*ext*_^*^, we obtain the partial derivatives w.r.t. the Cartesian coordinates *α* = *Δx, Δy, Δz*, using the property

$${\partial \over {\partial \alpha }}(1/{r^2}) = - 2\alpha /{r^4}$$

.

In this way we find that the components of the forces are

$$\eqalign{ & {F_x} = - 3\varepsilon _{ext}^*/r[\Delta xH_x^2/r\left\{ {\Delta {x^2}/{r^2} - 1} \right\} + \Delta x\Delta {z^2}H_z^2/{r^3} + \Delta z{H_x}{H_z}/r\left\{ {2\Delta {x^2}/{r^2} - 1} \right\}], \cr & {F_y} = - 3\varepsilon _{ext}^*/r[\Delta {x^2}\Delta yH_x^2/{r^3} + \Delta y\Delta {z^2}H_z^2/{r^3} + 2\Delta x\Delta y\Delta z{H_x}{H_z}/{r^3}], \cr & {F_z} = - 3\varepsilon _{ext}^*/r[\Delta {x^2}\Delta zH_x^2/{r^3} + \Delta zH_z^2/{r^3} + \Delta zH_z^2/r\left\{ {\Delta {z^2}/{r^2} - 1} \right\} + \Delta x{H_x}{H_z}/r\left\{ {2\Delta {z^2}/{r^2} - 1} \right\}] \cr} $$

.

The forces *F* and −*F* are now applied to particles 1 and *n* of the mesogen.
